# Factors associated with premature mortality among young injection drug users in Vancouver

**DOI:** 10.1186/1477-7517-4-1

**Published:** 2007-01-04

**Authors:** Cari L Miller, Thomas Kerr, Steffanie A Strathdee, Kathy Li, Evan Wood

**Affiliations:** 1 British Columbia Centre for Excellence in HIV/AIDS, St. Paul's Hospital, Vancouver, Canada; 2 University of British Columbia, Department of Medicine, Vancouver, Canada; 3 University of California at San Diego, Division of International and Cross-Cultural Medicine, San Diego, USA

## Abstract

**Background:**

Young injection drug users (IDUs) may be at increased risk of premature mortality due to the health risks associated with injection drug use including overdoses and infections. However, there has been little research conducted on mortality causes, rates and associations among this population. We undertook this study to investigate patterns of premature mortality, prior to age 30 years, among young IDUs.

**Methods:**

Since 1996, 572 young (≤29 years) IDUs have been enrolled in the Vancouver Injection Drug Users Study (VIDUS). Semi-annually, participants have completed an interviewer-administered questionnaire and have undergone serologic testing for HIV and hepatitis C (HCV). Mortality data have been continually updated through linkages with the Provincial Coroner's Office. Crude and age-specific mortality rates, standardized mortality ratios, and life expectancy measures were calculated using person-time methods. Predictors of mortality were identified using Cox regression analyses.

**Findings:**

Twenty-two participants died prior to age 30 years during the follow-up period for an overall crude mortality rate of 1,368 per 100,000 person-years. Overall, young IDUs were 16.4 times (95% confidence interval [CI]; 9.1–27.1) more likely to die; young women IDUs were 54.1 times (95%CI; 29.6–90.8) and young men IDUs were 12.9 times (95%CI; 5.5, 25.3) more likely to die when compared to the Canadian non-IDU population of the same age. The leading observed cause of death among females was: homicide (N = 9); and among males: suicide (N = 3) and overdose (N = 3). In Cox regression analyses, factors associated with mortality were, HIV infection (Hazard Ratio [HR]: 4.55; CI: 1.92–10.80) and sex work (HR: 2.76; CI: 1.16–6.56).

**Interpretation:**

Premature mortality was 13 and 54 times higher among young men and women who use injection drugs in Vancouver than among the general population in Canada. The majority of deaths among the women were attributable to homicide, suggesting that interventions should occur not only through harm reduction services but also through structural interventions at the legal and policy level.

## Background

Premature mortality among injection drug users (IDUs) is higher than in the general population with rates of mortality estimated to range between 0.8–3.26/100 person-years [1,2]. Young IDUs are at higher risk for a number of adverse health outcomes, including blood-borne infection, than among young people in the general population[3]. In a study of new onset injection drug users, mortality rates varied by calendar year, were elevated in comparison to the general population and were estimated to be 3.3 per 100-preson years [2]. In 2002, Roy et al. reported that street youth in Montreal, Quebec, aged 29 years and younger, had a standardized mortality ratio of 11.4 and one of the independent predictors of mortality was injection drug use [4]. Younger IDUs represent an important group to examine with respect to mortality due to their higher risk for drug related harms [5,6] and the opportunity to offer new information regarding avenues for prevention among this vulnerable population.

Recent studies in the United States and Scotland have found that mortality rates peaked among IDUs in the mid-1990s due to an increase in HIV/AIDS related deaths and have since declined [2,7]. Mortality among IDUs typically result from infectious diseases, overdose and injuries [8-10]. Overdose is a leading cause of death among IDUs [11] and varies between calendar years depending on factors such as purity and quality of drug availability and potentially on the HIV status among individuals [12,13]. Among IDUs in Edinburgh, Scotland deaths due to overdose and suicide were higher among younger IDUs than among older IDUs, with higher proportions of young males than females dying by suicide [7]. In the study of street youth in Montreal, Quebec, overdose deaths and suicide represented the leading causes of premature mortality [4].

Investigating causes of mortality among IDUs is important not only as a means for understanding risk among this population, but mortality can also be a measure of how well existing public health interventions are working to address drug-related harms. Studies have shown increased mortality rates since the advent of AIDS among IDUs, particularly prior to the advent of HIV antiretroviral therapy [7,14]. Nevertheless, other studies have shown that IDUs are more likely to die without ever accessing lifesaving HIV treatment when compared to other populations affected by HIV [15]. This information provides public health agencies with knowledge regarding a gap in the scope and effectiveness of existing systems of care. Thus, information on mortality can provide critical public health information for authorities to gauge how well existing services have been effective in addressing the ongoing public health crisis among IDUs.

This study was designed to investigate factors associated with mortality prior to age 30 years among IDUs and to determine rates and causes of premature mortality in this population.

## Methods

### Study population

The Vancouver Injection Drug User Study (VIDUS) is a prospective study of IDUs who have been recruited through self-referral and street outreach from Vancouver's Downtown Eastside (DTES) since May 1996. To date there have been over 1600 IDUs enrolled, among whom over 500 are young (aged ≤29 years). The Downtown Eastside is Vancouver's poorest neighborhood where an estimated 4,700 IDUs and 1,000 street youth reside in an area of approximately ten city blocks, and where inexpensive housing in the form of hotels and single room occupancies (SROs) are abundant. The cohort has been described in detail previously [16]. Briefly, persons were eligible for this study if they had injected illicit drugs at least once in the previous month confirmed by track site inspection, were aged 14 years and older and resided in the greater Vancouver region. At baseline and semi-annually, subjects provided venous blood samples and completed an interviewer-administered questionnaire. All participants provided informed consent, and were given a stipend ($20 CDN) at each study visit. The study has been approved by the University of British Columbia's Research Ethics Board.

### Sources of information on cause of death

The VIDUS office is situated in the hub of the DTES and the office serves as a drop-in where participants regularly stop by for coffee and conversation. Many of the VIDUS staff have been working in the community for several years and stay connected with residents and other community workers. This close community serves as an informal watch where information is shared when residents become missing, ill, incarcerated or die. This informal system is complemented by regular linkages with the provincial Coroner's Office where the coroner's report is reviewed for each confirmed death within the study. In addition, the provincial Vital Statistics Agency is reviewed to confirm deaths among participants twice annually. Thus, information on cause of death were obtained through regular follow-up, coroner's reports, and annual electronic linkages with BC Vital Statistics. These methods help to ensure the accuracy of information and avoid potential under representation due to reporting delays. The underlying cause of death reported on each death record was coded in accordance with the International Classification of Diseases, Tenth Revision (ICD-10).

### Statistical analyses

Socio-demographic variables included in these analyses were gender, ethnicity (Aboriginal vs. other) [17], HIV and HCV-positivity and homelessness. Aboriginal is self-reported and includes: First Nations people, Inuit and/or Métis people. Homelessness was defined as sleeping in the street, shelter and/or squat. Drug and sexual risk variables included in these analyses were history of sexual abuse, sex work, greater than once daily crack cocaine use and greater than once daily injection of heroin, cocaine and/or speedball (a mixture of heroin and cocaine), and use of methadone maintenance therapy (MMT). Sex-work involvement was defined as exchanging sex for money, goods, drugs, or shelter. All time-updated variables refer to activities in the six months prior to each semi-annual follow-up visit with the exception of sexual abuse, defined as ever occurring.

Baseline characteristics are described in Table 1 and causes of death are described in Table 2. For the longitudinal analyses, Table 3, the follow-up period for each participant started at baseline and ended at the first of the following events: death or age 30 years. Mortality rates were calculated overall and by subgroups defined by variables selected from the above listed characteristics, based on the literature and appropriateness for the sample size. Mortality rates were calculated using the person-time method (18); 95% confidence intervals (CI) were calculated using the Poisson distribution.

**Table 1:** Characteristics of the 572 young (≤29 years) Vancouver injection drug user study participants at baseline.

**Characteristic**	**No. (%)**
Females	268 (47%)
Aboriginal	163 (29%)
HIV Positive at Baseline	92 (16%)
HCV Positive at Baseline	326 (57%)
Homeless in the 6 mos. prior	144 (25%)
Sex Abuse Ever	231 (40%)
Sex Work in the 6 mos. prior	252 (44%)
≥1 per day Crack in the 6 mos. prior	57 (10%)
≥1 per day Heroin in the 6 mos. prior	260 (45%)
≥1 per day Cocaine in the 6 mos. prior	188 (33%)
≥1 per day Speedballs in the 6 mos. prior	78 (14%)
Methadone Maintenance Therapy in the 6 mos. prior	31 (5%)

**Table 2:** Profile of cause of death among young (≤29 years) injection drug users in Vancouver who died between 1996 and 2006 (N = 22).

	Cause of Death	Females No.	Males No.	Total
	Homicide	9		9
	Accident	1	1	2
	Suicide		3	3
	HIV	2	1	3
	Overdose	1	3	4
	Undetermined Illness	1		1
	Total No.	14	8	22

**Table 3:** Mortality rates and cox regression analyses of mortality among young (≤29 years) injection drug users (N = 572) in Vancouver between 1996 and 2006.

**Characteristic**	**No. of Deaths**	**Mortality Rate per 100,000 Person Years**	**Unadjusted Hazard Ratio (95% CI)**	**Adjusted Hazard Ratio (95% CI)**
Older than 24 yrs.				
Yes No	13 9	1,679 1,213	1.41 [0.60–3.30]	
Female				
Yes No	14 8	1,645 1,057	1.77 [0.74–4.22]	
Aboriginal				
Yes No	7 15	1,282 1,412	1.07 [0.44–2.62]	
HIV				
Yes No	13 9	3,137 1,035	4.55 [1.92–10.80]	4.01 [1.67–9.56]
HCV				
Yes No	15 7	1,689 959	0.96 [0.37–2.51]	
Homelessness				
Yes No	5 17	1,220 1,412	1.19 [0.44–3.25]	
Sex Work				
Yes No	16 6	2,159 692	2.76 [1.16–6.56]	1.97 [0.80–4.84]
Sexual Abuse				
Yes No	12 10	1,829 1,050	1.66 [0.72–3.84]	
≥1 per day Heroin				
Yes No	10 12	1,389 1,351	0.84 [0.35–1.97]	
≥1 per day Cocaine				
Yes No	8 14	1,501 1,302	1.40 [0.58–3.37]	
≥1 per day Crack				
Yes No	5 17	2,959 1,181	2.41 [1.00–5.81]	1.94 [0.79–4.80]

Standardized mortality ratios were calculated using the indirect method of standardization by sex and age group. The comparison group was the Canadian population of the same age in 2000. Abridged life tables were calculated using methods adopted by Lopez et al. at the World Health Organization [19]. Predictors of mortality were identified using univariable and multivariable Cox regression analyses. All variables with p values ≤ 0.05 in univariable analyses were included in multivariable analyses.

## Results

### Characteristics of the study participants

Between May 1996 and December 2004, 572 participants aged ≤ 29 years were enrolled into the study. Participants completed between 1 and 15 questionnaires (average 7 per participant; 83% completed at least 1 follow-up questionnaire following the baseline interview). During follow-up 182 participants reached 30 years of age. In total, participants accumulated 1608 person-years of follow-up time prior to age 30 years.

The median age of participants at study entry was 23.9 (IQR: 20.9–26.3) and the number of years injecting was 4 (IQR: 1.5–8). As indicated in Table 1, 47% were female and 29% were of Aboriginal ancestry. The percentage of young people HIV and HCV infected was 16% and 57% respectively and 25% were homeless. Of the sex risk variables, 40% reported a history of sexual abuse and 44% engaged in sex work. Among the young participants, 10% had smoked crack daily, 45% had injected heroin daily, 33% had injected cocaine daily, 14% had injected speedballs (heroin and cocaine combined) daily and 5% had accessed methadone maintenance therapy (MMT).

### Mortality

In total, 42 deaths occurred during the study period, 20 of those occurring after 30 years of age and were excluded from further analyses. Thus, there were 22 deaths that occurred during the follow-up period among participants aged 29 years and younger. Of note, 1 of the observed deaths was classified as "assault" and for this study we included it in the homicide category. Thus, among females, the leading cause of death (refer to Table 2) was homicide (n = 9) and among males, suicide (n = 3) and overdose death (n = 3). Death due directly to HIV infection occurred among 2 female participants and 1 male participant.

The 22 deaths observed among this population during follow-up represented a mortality rate of 1368 per 100,000 person-years. Among females, the mortality rate was 1645 per 100,000 person-years and among males, the rate was 1045 per 100,000 person years. In comparison with the Canadian population of the same age in 2000, young IDUs were 16.4 times (95% confidence interval [CI]; 9.1–27.1) more likely to die; women were 54.1 times (95%CI; 29.6–90.8) and men were 12.9 times (95%CI; 5.5, 25.3) more likely to die. At age 15, IDUs could expect to live another 36.8 years, compared to the Canadian population at age 15 who could expect to live another 64.8 years or nearly double the life expectancy of IDUs in this study population.

Univariable and multivariable Cox regression analyses assessing associations between mortality and participant characteristics are presented in Table 3. In univariable analyses, factors associated with mortality among the study population were sex work (Hazard Ratio [HR]; 2.76 [95%CI; 1.16–6.56]) and HIV infection (HR; 4.55 [95%CI; 1.92–10.80]). The only factor to remain significantly associated with mortality among participants in multivariable analyses was HIV infection (HR; 4.55 [95%CI; 1.92–10.80]).

## Discussion

The mortality rate observed among this population of young people is high. Young male and female IDUs in this setting had rates of mortality that were 12 and 51 times higher respectively than the Canadian population of the same age. Life expectancy at age 15 years is half of what is observed at a national level. Particularly concerning was the number of deaths due to homicide among the women in the study.

A previous study identified mortality from homicide as the leading cause of death among young homeless males and females in an urban setting in the United States where homicide rates are generally higher than in other developed nations[20]. However in this Canadian setting where homicide deaths rank low, young drug dependent women appear to be at very high risk of death by this means. The high number of women dying by homicide combined with the generally low rate of homicide in this setting warrants public health intervention, particularly due to the preventable nature of this cause of death. In this study, approximately half of the participants were involved in sex work at baseline and among females, this figure approaches 80% (data not shown). In longitudinal analysis, sex work was an important predictor of mortality in this study, however this factor did not reach significance in multivariable analyses likely due to power issues. The relationship between injection drug dependency, younger age, female sex and sex work has previously been shown [21-24].

Of note, investigation of Robert Pickton for the serial murders of drug dependent women from Vancouver's Downtown Eastside has recently begun [25]. This investigation may account for the high number of homicide deaths observed among women in this setting. Other similar investigations in parts of Mexico and the US (Ciudad Juarez and the Green River serial killer investigations) suggest that women, and particularly young women, who engage in sex work are at high risk for being targeted by sexual predators [26,27]. It has also been suggested by community workers that young women who deal drugs to support their habits may rank low in the hierarchy of drug dealing relationships and may be at risk for death by "being made an example of" when using the drugs they are meant to sell. The development of public health interventions to reduce the risk for violence among young injection drug dependent women who engage in sex work is important. More recently, legal reform for sex workers in this setting has been proposed and these findings underscore the need to support legal reform and other harm reduction initiatives for sex workers to reduce the risk of violence and homicide death[28]. Additional public health interventions require further investigation, particularly qualitative, to ascertain types of interventions that may be acceptable to young female IDUs who also engage in sex work. Given the potentially deadly consequences, considering innovative drug treatment and pharmaco-therapeutic interventions, such as prescription drug maintenance, may help to reduce drug-related harms, including premature mortality, in this population [29].

In the final Cox model, the only predictor of premature mortality was HIV infection. Similarly, Roy et al. found that HIV was the strongest predictor of mortality among Montreal street youth; however HIV represented a small proportion of the overall causes of death [4]. The consistency between these results may imply that youth who are vulnerable to premature mortality are also those more vulnerable to blood-borne infections.

Similar to other findings regarding mortality among younger age groups and males in particular, death by suicide and overdose were common[30]. In this study, the deaths by overdose were not deemed intentional by coroner reports, however other literature has indicated that overdose may be one of the ways that young people commit suicide and among IDUs, intentional suicide by overdose may be may be hard to prove[30]. Suicide among young people is always a tragic phenomenon and given the higher risk for suicide, community suicide prevention resources should be mobilized within this population[31]. In addition, ensuring overdose prevention education and available tools are accessible to younger IDUs may be important for prevention of premature mortality in this population.

There are several limitations that should be considered with regards to the data presented here. First, this study sample was relatively small and although a smaller number of associations were considered, power issues may have constrained the longitudinal analyses. The second limitation may be the potential for misclassification bias relating to self-reported behaviours, however the interviewers are trained to probe for any misleading information and every precaution is taken to assure the participant of confidentiality. Third, there is a possibility that the number of deaths occurring were underestimated, particularly if the participant was lost to follow-up or the death occurred out-of-province. Finally, in this setting, a higher number of homicides were found among young women than in other studies suggesting that these results may represent an anomaly. However, the experiences of sex workers who work without legal protection, such as in most North American settings and other settings worldwide, violence and the risk of predation is high and for drug dependent women, the risks may be even greater[32]. There is a need for more research on violence and predation among young women involved in sex work and a need for better protection of their human rights.

Mortality among IDUs may be an assumed risk consequential to a high-risk behaviour. However the data presented here suggests that the majority of risk for premature mortality among young IDUs is resulting, not directly from injection drug use, but indirectly from preventable causes. Clearly, better public health interventions must be implemented targeting this population including emergency and long term housing options, alternative employment training for young sex workers and accessible substitution therapies for young IDUs. In addition, given the ongoing harms associated with sex work, structural changes including legal and policy reform are warranted. The high rates of mortality presented here should send a clear message to public health agencies that young IDUs have unique risk profiles and innovative interventions are required to avert preventable premature mortality among this population.
